# The role of exosomes in lung cancer metastasis and clinical applications: an updated review

**DOI:** 10.1186/s12967-021-02985-1

**Published:** 2021-07-19

**Authors:** Lei Yin, Xiaotian Liu, Xuejun Shao, Tao Feng, Jun Xu, Qi Wang, Shenghao Hua

**Affiliations:** 1 Clinical Laboratory, Children’s Hospital of Soochow University, Suzhou, 215000 People’s Republic of China; 2 Department of Anesthesiology, Children’s Hospital of Soochow University, Suzhou, 215000 People’s Republic of China

**Keywords:** Lung cancer, Exosome, Metastasis, Clinical application, Biomarker, Diagnosis, Drug resistance, Therapy

## Abstract

Lung cancer is the leading cause of cancer-associated deaths accounting for 24% of all cancer deaths. As a crucial phase of tumor progression, lung cancer metastasis is linked to over 70% of these mortalities. In recent years, exosomes have received increasing research attention in their role in the induction of carcinogenesis and metastasis in the lung. In this review, recent studies on the contribution of exosomes to lung cancer metastasis are discussed, particularly highlighting the role of lung tumor-derived exosomes in immune system evasion, epithelial-mesenchymal transition, and angiogenesis, and their involvement at both the pre-metastatic and metastatic phases. The clinical application of exosomes as therapeutic drug carriers, their role in antitumor drug resistance, and their utility as predictive biomarkers in diagnosis and prognosis are also presented. The metastatic activity, a complex multistep process of cancer cell invasion, survival in blood vessels, attachment and subsequent colonization of the host's organs, is integrated with exosomal effects. Exosomes act as functional mediating factors in cell–cell communication, influencing various steps of the metastatic cascade. To this end, lung cancer cell-derived exosomes enhance cell proliferation, angiogenesis, and metastasis, regulate drug resistance, and antitumor immune activities during lung carcinogenesis, and are currently being explored as an important component in liquid biopsy assessment for diagnosing lung cancer. These nano-sized extracellular vesicles are also being explored as delivery vehicles for therapeutic molecules owing to their unique properties of biocompatibility, circulatory stability, decreased toxicity, and tumor specificity. The current knowledge of the role of exosomes highlights an array of exosome-dependent pathways and cargoes that are ripe for exploiting therapeutic targets to treat lung cancer metastasis, and for predictive value assessment in diagnosis, prognosis, and anti-tumor drug resistance.

## Background

Regardless of the advances made in our understanding of risk, development, immunologic control, and treatment options, lung cancer remains the leading cause of cancer death globally [1, 2]. Metastasis is a major cause of death in lung cancer patients, during which cancer cells spread to new areas of the body, usually through the bloodstream or lymphatic systems. The metastatic process, from initial primary tumor growth through angiogenesis and intravasation, to survival in the bloodstream, extravasation, and metastatic growth, is an inefficient series of steps, and a few released cancer cells complete the entire process. Microenvironmental interactions and associated factors such as exosomes and their cargos contribute to each of these steps to ensure successful tumor migration and invasion, hence the discovery of mechanisms by which cancer cells interact with the microenvironment could contribute to uncover key molecules, such as biomarkers or potential drug targets [3, 4]. It has been demonstrated that cancer cells secrete approximately tenfold more exosomes than normal cells and that these tumor-derived exosomes facilitate cellular communication via delivery of growth factors, chemokines, RNAs, proteins, and lipids [5–7]. Tumor-derived exosomes participate in the formation of a pre-metastatic niche which plays an important role in the metastatic process by preparing an appropriate microenvironment in specific organs for tumor metastasis. In effect, exosomes are key contributors to the formation and remodeling of the lung cancer microenvironment by enhancing immune cell evasion, epithelial-mesenchymal transition (EMT), and angiogenesis, aimed at increasing the metastatic ability of lung cancer cells [8].

Exosomes are 30–150 nm membrane vesicles that are produced in the endosomal compartment of cells and play a role in intercellular modulation of both physiological and pathological activities [9]. As a subset of extracellular vesicles, the biogenesis of exosomes involves their origin in endosomes, and subsequent interactions with other intracellular vesicles and organelles produce the final content of the exosomes [10]. They are actively released by cells via an exocytosis pathway during crosstalk between cells and in receptor uptake mechanisms. This pathway involves initiation of activated growth factor receptors located on the plasma membrane surface [11, 12]. Exosomes from different sources share common exosomal constituents and are composed of varying quantities of macromolecules mainly proteins, messenger RNAs (mRNAs), microRNAs (miRNAs), and lipids [10, 13]. In addition to tumor-derived exosomes, many other sources of exosomes include stem cells, immune cells, body fluids (such as blood, amniotic fluid, saliva, urine, and breast milk), intestinal epithelial cells, and food [9].

The high mortality of lung cancer is attributed to the fact that most cases are diagnosed at the advanced stage when metastasis might have already taken place, offering limited treatment options and 5‐year survival rates of approximately 4%. Therefore, the identification of reliable predictive biomarkers for diagnosis and prognosis, as well as effective therapeutic targets and strategies is an unmet medical need in lung cancer [14, 15]. Interestingly, researchers have shown that tumor-derived exosomes possess unique miRNA and mRNA expression profiles that may differ from healthy individuals, hence a promising predictive biomarker for lung tumors [16, 17]. Moreover, exosomes can be engineered as delivery vehicles for transferring functional biomolecules, such as nucleic acids (DNA, miRNA, mRNA), proteins, lipids, and other drugs to target sites to influence inflammation, apoptosis, angiogenesis, and metastasis. Several studies have demonstrated the effective application of exosomes as therapeutic carriers in many conditions such as cancer [18], inflamed brain [19], Parkinson’s disease [20], among others [9, 21–23].

In the background that tumor metastasis often indicates poor prognosis and remains the leading cause of cancer-associated death, meaningful studies providing further insight in this process are crucial to identifying preventive and diagnostic targets. In this paper, recent data on the contribution of exosomes in lung cancer metastasis are discussed, particularly highlighting the role of lung tumor-derived exosomes in immune evasion, EMT, and angiogenesis, and involvement at both the pre-metastatic and metastatic phases. The clinical application of exosomes in diagnosis, prognosis, drug resistance, and therapeutics are also presented.

## General exosomal functions that aid lung cancer metastasis

Exosomes expressed in the tumor microenvironment are known to actively modulate the activity of recipient cells, and contribute to the process of tumor growth and metastasis by participating in cellular communications, regulating cell signaling, and encouraging the formation of a pre-metastatic niche [24, 25]. Key outcomes of these modulations include enhanced immune system evasion, increased angiogenesis, and induced EMT, consequently assisting lung cancer cells to metastasize.

### Immune system evasion in lung cancer

Tumor-derived exosomes transfer immunosuppressive molecules via direct contact or paracrine signaling to immune cells, leading to suppressed functions and promotion of tumor progression [26]. To efficiently evade the host immune system, the tumor derives exosomes to modulate antitumor immune responses via the inhibition of T-cell activation and proliferation, induction of regulatory T-cells (Tregs) and myeloid-derived suppressor cells (MDSCs), and inhibition of natural killer (NK) and CD8 + T-cells function, thus facilitating tumor progression and metastasis [27, 28]. Furthermore, exosomes from lung cancer cells, melanoma, and breast cancer cells carry immunosuppressive programmed death-ligand 1 (PD-L1), which binds to PD-1 through its extracellular domain to inactivate T cells. The expression of exosomal PD-L1 is upregulated by interferon-γ (IFN-γ), which causes the suppression of CD8 + T cell function and facilitates tumor growth [29]. Other studies have demonstrated that exosomal PD-L1 enables tumor cell survival, hence genetic blockade or antibody inhibition of exosomal PD-L1 can facilitate T-cell activity in the draining lymph node, improving systemic antitumor immunity and memory [30]. These exosomes also impair immune functions by reducing cytokine production and inducing apoptosis in CD8 + T cells [31]. It has been reported that hypoxia preconditioned tumor-derived microvesicles produce transforming growth factor (TGF)-β1 and miR-23a that can inhibit the cytotoxicity of NK cells in vitro and in vivo [32]. Another study found that approximately 80% of exosomes purified from lung cancer biopsies contained endothelial growth factor receptor (EGFR), which was present in only 2% of exosomes obtained from chronic lung inflammation samples. The purified exosomes induced tolerogenic dendritic cells (DCs). Further analysis indicated that co-culture of the tolerogenic DCs and Th0 cells produced tumor antigen-specific Treg, which could inhibit the tumor antigen-specific CD8 + T cell functions [33]. These findings collectively show that tumor-derived exosomes can rescue tumor cells by evading immune cell surveillance, presenting a therapeutic target that could contribute to the development of immunotherapeutic approaches for cancer therapy.

### Epithelial-mesenchymal transition in lung cancer

**Fig. 1:**
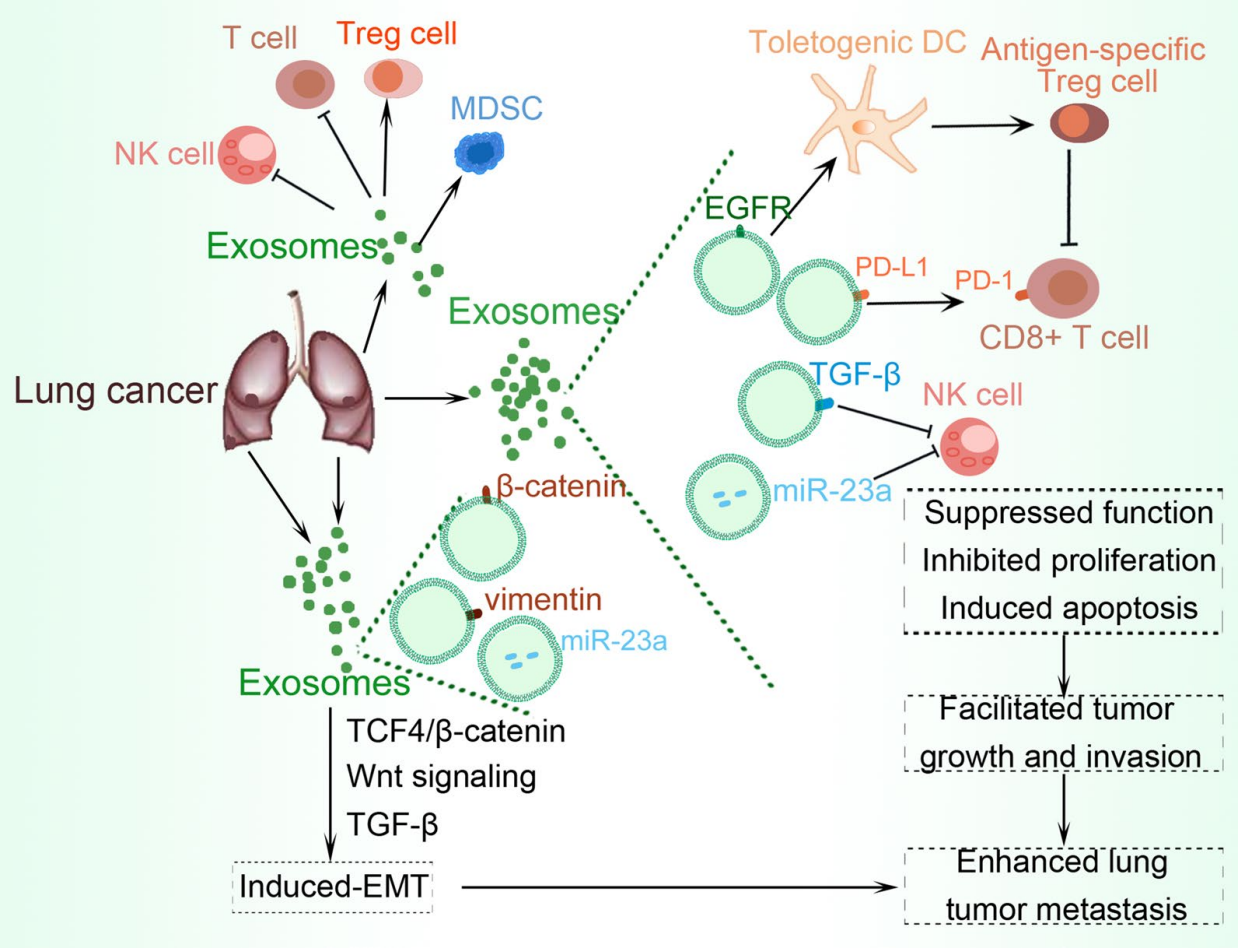
The role of exosomes in immune evasion and EMT promotion in lung cancer. In the lung cancer tumor microenvironment, there is increased expression of exosomes which inhibit tumor-cytotoxic cells such as NK and T cells, while enhancing the recruitment, activation, and expansion of immunosuppressive cells such as MDSCs and Tregs. Exosomal cargoes including EFGR, PD-L1, TGF-β, miR-23a, Wnt, and their associated signaling, facilitate immune evasion and induce EMT, resulting in tumor metastasis

EMT, a highly conserved process that favors tumor cell migration and invasion occurs due to the loss of cell–cell adhesion properties, and the resultant acquisition of the mesenchymal phenotype. Evidence from several recent studies indicates that exosomes are key contributors to the EMT process, during which they mediate the transfer of mesenchymal-associated information between cancer cells and their microenvironment, and modulate signal transduction in recipient cells [34, 35]. Exosomes obtained from TGF-β1-treated mesenchymal cells, exhibited upregulated level of β-catenin but decreased expression of E-cadherin and vimentin. Moreover, miR-23a was significantly increased in the secreted exosomes. Further analysis indicated that exosomes activated TCF4/β-catenin transcriptional activity and initiated canonical Wnt signaling in A549 cells undergoing EMT [36]. The exosomal profile of small RNAs alters following EMT, and these specific miRNAs potentially drive signal transduction networks in EMT and cancer progression [37]. Exosomes derived from highly metastatic lung cancer cells, PC14HM, express a higher levels of vimentin than those from non-metastatic lung cancer cells, PC14. Treatment of human bronchial epithelial cells with PC14HM-derived exosomes, resulted in a significantly increased level of vimentin, triggering EMT in recipient HBECs [38]. These results suggest tumor-derived exosomes to be key drivers of EMT via the transformation of tumor cells to a more aggressive phenotype. Figure 1 summarizes the EMT and immune evasion activities of these exosomes.

### Angiogenesis in lung cancer

**Fig. 2:**
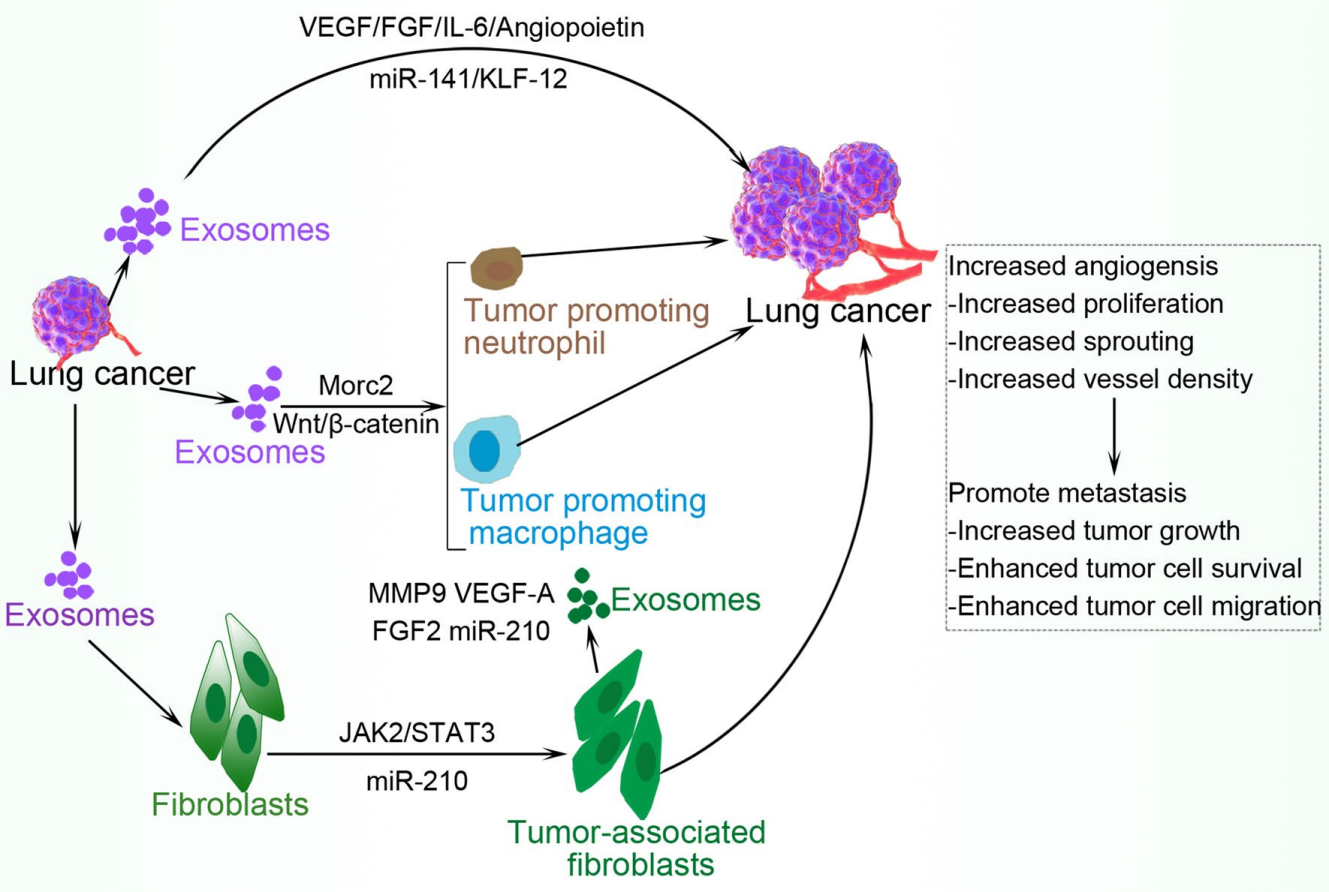
Exosomal-induced angiogenesis in lung tumor. Lung tumor-derived exosomes induce fibroblasts to secrete exosomes that express factors such as MMP9, VEGF-A, FGF2, and miR-210 via the JAK2/STAT3 signaling pathway. These exosomes together with tumor-produced vesicles enhance the activation of tumor-promoting cells such as macrophages and neutrophils. Other exosomal cargoes directly trigger angiogenesis by increasing blood vessel proliferation, sprouting, and density, resulting in promoted metastasis

Exosomes induce tumor-associated angiogenesis (Fig. 2), a process through which new blood vessels are formed from pre-existing vessels by transferring or expressing protein molecules such as vascular endothelial growth factor (VEGF), fibroblast growth factors (FGF), interleukin (IL)-8, IL-6, and angiopoietin [7, 39, 40]. Angiogenesis is a basic requirement for the survival of tumor cells and is considered to be a malignant feature of small cell lung cancer (SCLC) which is closely linked to the poor prognosis of SCLC patients. Exosomes produced by lung cancer cells promote angiogenesis as evidenced by enhanced human umbilical vein endothelial cells (HUVECs) proliferation and tube formation, as well as inhibited apoptosis of HUVECs. Conversely, growth arrest-specific 5 (GAS5) competitively bound miRNA-29-3p with phosphatase and tensin homolog (PTEN), leading to upregulated PTEN mRNA and protein expression, and suppressed levels of phosphatidylinositol-4,5-bisphosphate 3-kinase catalytic subunit alpha (PI3K) and serine/threonine kinase 1 (Akt) phosphorylation in HUVECs to inhibit lung tumor-associated angiogenesis [41]. Circulating miR-141 is upregulated in samples of SCLC patients and significantly correlates with advanced TNM stages. SCLC-derived exosomes deliver miR-141 to HUVECs via exosomes facilitating HUVEC proliferation, migration, invasion, and tube formation, causing enhanced microvessel sprouting from mouse aortic rings. Further, exosomal miR-141 trigger neoangiogenesis in vivo with higher microvessel density and growth via the miR-141/KLF12 pathway [42]. Cancer-associated fibroblasts (CAFs) capably increases the process of tumor angiogenesis. In one study, lung cancer-derived exosomes induced fibroblast reprogramming into CAFs, with the exosomes overexpressing miR-210 as well as other proangiogenic factors such as MMP9, VEGFA, and FGF2, thus stimulating an increased level of angiogenesis. Mechanistically, exosomal miR-210 enhanced the proangiogenic switch of CAFs through the regulation of JAK2 (Janus kinase 2)/STAT3 (signal transducer and activator of transcription 3) signaling pathway and TET2 in recipient fibroblasts [43]. MORC family CW-type zinc finger 2 (MORC2) promotes cancer progression by enhancing angiogenesis and recruitment of tumor-associated macrophage via the Wnt/β-catenin pathway in lung cancer [44].

## Exosomes and lung cancer metastasis

As the main cause of lung cancer mortality, metastasis is of great concern in cancer research. Exosomes serve as key mediators of intercellular communication and are important components of the cancer microenvironment that contributes to cancer development, progression, and metastasis [45, 46]. Because of the unstable nature of oncogenes, factors such as inflammation, hypoxia, and acidosis can trigger tumor cells to secrete more exosomes to help form a tumor microenvironment that favors the rapid growth of tumor cells and enhances their ability for invasion and metastasis. The exosome-induced cancer cell migration is associated with increased expression of molecules like TGF-β and IL-10 in tumor-derived exosomes [47]. In addition to tumor derived-exosomes, the effects of exosomes derived from other sources such as the bone marrow, adipocytes, and human umbilical cord, among others has also been investigated.

### Tumor-derived exosome

#### Involvement in the pre-metastatic niche phase

The pre-metastatic niche educated by primary tumor-derived factors such as exosomes and host stromal cells contributes to cancer metastasis. The expression of niche-characteristic genes, such as BV8, MMP9, S100A8, and S100A9 have been implicated in pre-metastatic niche formation, as well as the promotion of tumor cell migration, invasion, and colonization in the metastatic site [48]. It has been shown that lung epithelial cells play a crucial role in the initiation of neutrophil recruitment and formation of lung metastatic niche by sensing tumor exosomal RNAs via toll-like receptor 3 (TLR3). In spontaneous metastatic mice models, TLR3-deficient mice showed decreased lung metastasis, while tumor-derived exosomal small nuclear RNAs activated TLR3 in lung epithelial cells to induce chemokine secretion (chemokine [C-X-C motif] ligand 1 [CXCL1], CXCL2, CXCL5, and CXCL12), and neutrophil recruitment [49]. Tumor-derived exosomes are capable of stimulating bone marrow-derived cell mobilization to form a pre-metastatic niche and even determine organotropic metastasis through the expression of membrane-bound integrins [50]. Certain exosomal integrins are linked with cancer cells of specific organs and predict organ-specific metastasis, such as exosomal integrins α6β1 and α6β4, which are associated with lung metastasis. This tumor-derived exosome uptake by organ-specific cells prepares the pre-metastatic niche. Moreover, the exosomal integrin uptake by resident cells activates Src phosphorylation and pro-inflammatory S100 gene expression [50].

Other studies that have investigated the role of tumor-derived exosomes in the formation of primary tumors and pre-metastases. In both mice and human subjects exosomes trigger tissue matrices remodeling and micro anatomic niche preparation that facilitate lymphatic metastasis by cancer cells [51]. Bone marrow-derived hematopoietic progenitor cells that express VEGFR1 home to tumor-specific pre-metastatic sites and form cellular clusters before the arrival of tumor cells [52], while the metastatic ability of primary tumors is increased by permanent exosomal education of bone marrow progenitor cells via the receptor tyrosine kinase MET [53]. Exosomes also trigger vascular leakiness at pre-metastatic sites to reprogram bone marrow progenitor cells toward a pro-vasculogenic phenotype with enhanced permeability of lung endothelial cells [53]. The RNAs RAB1A, RAB5B, RAB7, and RAB27A, regulators of exosome formation and membrane trafficking are significantly expressed in cells, with interference in Rab27A RNA levels causing reduced exosome production, tumor growth, and metastasis [53]. Similarly, the administration of B16-F10 exosomes into the lung tissue resulted in increased levels of genes involved in extracellular matrix remodeling and inflammation, including pre-metastatic niche formation molecules such as S100A9, and S100A8, and tumor necrosis factor α (TNFα) as a mediator of vascular permeability [54]. The exosomal miR-25-3p is involved in pre-metastatic niche formation by regulating the expression of VEGFR2, occludin, ZO-1, and Claudin5 in endothelial cells via targeting KLF2 and KLF4, and consequently promoting vascular permeability, angiogenesis, and metastasis [55]. Tumor-associated exosomes also promote angiogenesis and vascular leakage [56] and stimulate coagulation and thus increase adherence to circulating tumor cells [57].

Reduction in immune surveillance is another important process in the establishment of the pre-metastatic niche. Tumor-derived exosomes have been shown to participate in the suppression of innate immune responses by mobilizing myeloid-derived suppressor cells [58], activating tumor-favorable macrophages [59], and neutrophils [60], causing natural killer (NK) cell dysfunction via exposing NKGD ligands [61], and impede adaptive immune responses through the inhibition of antigen-presenting cells (APCs) and cytotoxic T cells through the blockade of their activation, proliferation, and enhancement of T cell apoptosis [62, 63]. In the lung tumor microenvironment, M0 macrophages internalize tumor-derived exosomes, leading to their differentiation into M2 phenotype, which exhibits anti-inflammatory and pro-tumoral effects [64].

#### Involvement in the metastatic phase

Tumor-derived exosomes influence lung cancer progression and metastasis by modulating the physiological activities of both surrounding tissue cells and microenvironmental factors. For example, tumor-associated exosomes trigger the transformation of mesenchymal stem cells (MSCs) into a pro-inflammatory phenotype through the nuclear factor-κ-B (NF-κB)/TLR signaling pathway to promote the acquisition of tumor supportive characteristics. Lung cancer-derived exosomes are enriched in signal transduction molecules such as EGFR, GRB2, and SRC [65], and functional exosomal RNAs such as circSATB2 and miR-660-5p [66, 67], that actively modulate recipient cells proliferation, and promote tumor growth, metastasis, as well as the abnormal proliferation of normal human bronchial epithelial cells. A study that investigated the regulatory mechanism of circ-CPA4, let-7 miRNA, and programmed cell death ligand 1 (PD-L1) in lung cancer reports that circ-CPA4 regulates cell growth, stemness, mobility, and drug resistance in non-small-cell lung carcinoma (NSCLC) cells and inactivates CD8 + T cells in the tumor microenvironment via the let-7 miRNA/PD-L1 axis. Conversely, the knock-down of circ-CPA4 inhibits cell growth, mobility, and EMT, while enhancing cell death in NSCLC cells by downregulating PD-L1, which serves as an RNA sponge for let-7 miRNA [68].

TGF-β has been shown to mediate exosomal miRNAs regulation of the migration and invasion of lung cancer cells. Pretreatment of lung cancer cells with TGF-β results in increased migration, vascular permeability, and invasive potential of lung cancer cells via TGF-β-mediated exosomal carriage of intercellular communication. In this process, exosomal long noncoding-matrix metalloproteinase 2 (lnc-MMP2-2) RNA regulates the migration and invasion of lung cancer cells by enhancing MMP2 expression [69]. Similarly, lnc-RNAs AC026904.1 and XIST are crucial in the promotion of TGF-β-induced migration and EMT by functioning as an enhancer of SLUG in lung cancer cells [70], and by modulating the miR-367/141-ZEB2 axis in NSCLC [71]. A study confirmed that miR-378 is significantly differentially expressed in NSCLC cells of patients with brain metastasis, and promotes cell migration, invasion, and tumor angiogenesis [72]. Exosomal miR-619-5p is also identified as a potent inducer of lung cancer-associated angiogenesis by targeting and suppressing RCAN1.4. The targeted inhibition of RCAN1.4 not only induces angiogenesis but also cell proliferation and metastasis in NSCLC cells [73].

Bone metastasis, occurring in about 20–40% of lung cancer patients, is the most frequent complication in NSCLC resulting in osteolytic lesions through the lost balance between bone-forming osteoblasts and bone-resorbing osteoclasts activity. In this process, the epidermal growth factor receptor (EGFR) pathway is constitutively activated and bound to amphiregulin. Lung cancer-derived exosomes express a high quantity of amphiregulin that contributes to the induction of bone metastasis. Mechanistically, the NSCLC-derived exosomes produce amphiregulin which induces EGFR pathway activation in pre-osteoclasts, consequently causing increased expression of receptor activator of nuclear factor-kappa-Β ligand (RANKL), associated with the vicious cycle in osteolytic bone metastasis [74]. Lung tumor-derived exosomal induction of osteoclastogenesis is also linked with factors such as acceleration by C-X-C chemokine receptor type 4 (CXCR4) through self-potentiation and vascular cell adhesion molecule 1 (VCAM1) secretion [75], differential expression of the RANKL/RANK/OPG system [76], and activation of the EGF pathway by multiple myeloma-derived exosomes enriched in amphiregulin in the bone microenvironment [77]. Sequencing of plasma-derived exosomal miRNAs of lung cancer patients revealed three consensus clusters that showed significant differential expression. Further analysis indicated that the cluster is likely involved in preconditioning the metastatic niche and promoting EMT and bone metastasis. The exosomal miR-574-5p, an inhibitor of the Wnt/β-catenin pathway, and miR-423-3p and miR-328-3p, activators of Wnt/β-catenin pathway were downregulated and upregulated in bone metastasis patients respectively [78].

SCLC has a strong predilection for early brain metastases, the main contributor to its mortality. Co-culture of SCLC cells with human brain microvascular endothelial cells (HBMECs) results in increased expression of S100A16, a protein-encoding gene associated with SCLC brain metastases. Exosomal mediated transfer and expression of S100A16 in recipient SCLC cells caused increased survival of the recipient SCLC cells, inhibited the loss of mitochondrial membrane potential, and encouraged cellular resistance to apoptosis under stressful conditions through prohibitin (PHB)‐l [79]. Exosomal cargoes that differentiate patients with and without brain metastasis in lung cancer have also been reported. There are higher levels of serum miR-330-3p in NSCLC patients with brain metastasis than those without metastasis, and its overexpression promotes proliferation, migration, invasion, and EMT of NSCLC in vivo and in vitro [80].

**Fig. 3:**
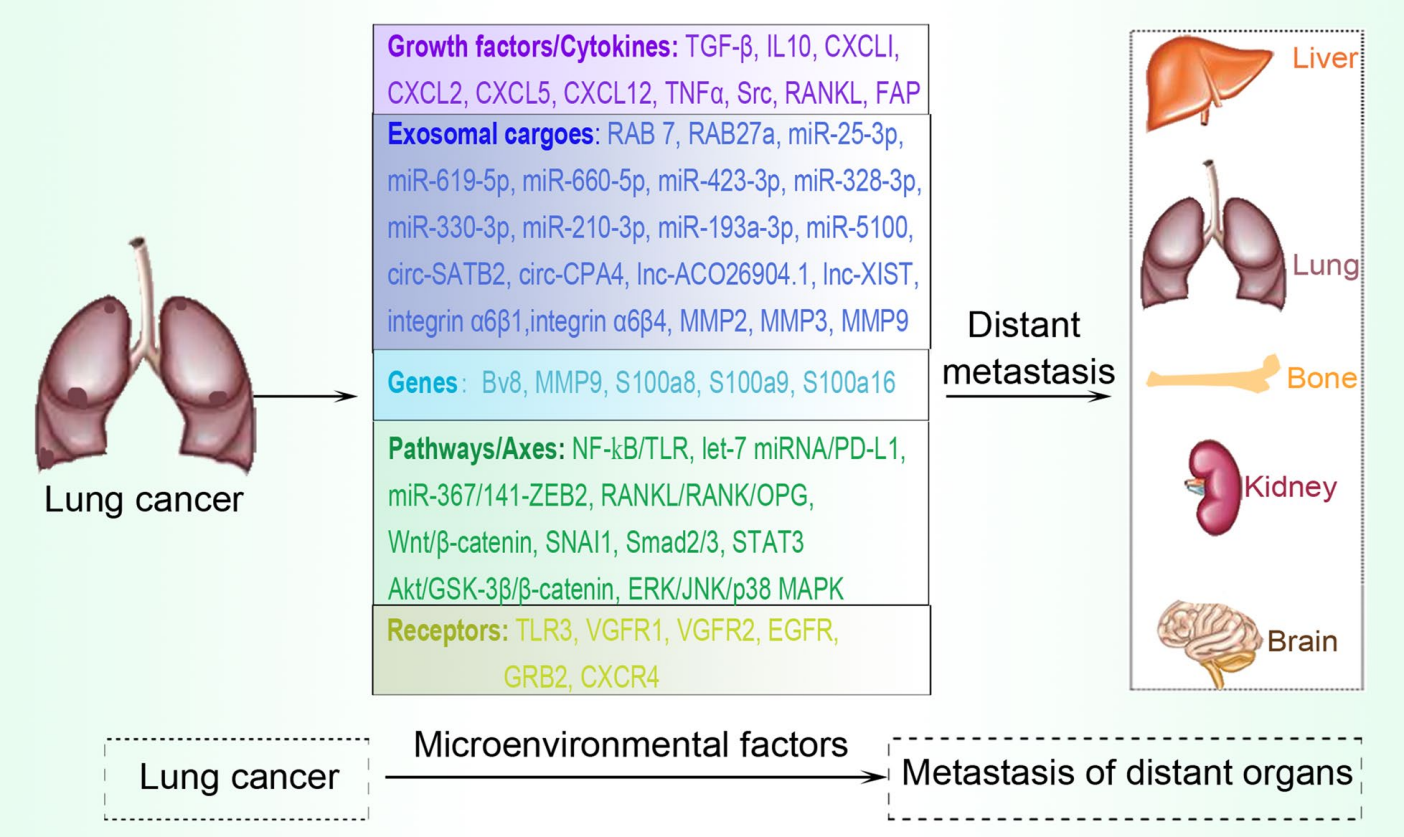
Lung tumor microenvironmental factors associated with exosomal metastatic promoting effects. Several factors, classified as growth/cytokine factors, exosomal cargoes, genes, pathways/axes, and receptors are directly or indirectly influenced by exosomes to participate in the promotion of lung tumor metastasis. Distant organs commonly prone to lung tumor metastasis include the liver, bone, kidney, brain, and other the contralateral unaffected lung

Tumor-derived exosomes contribute to the formation of invadopodia, actin-rich protrusions of the plasma membrane that are linked with degradation of the extracellular matrix in cancer invasiveness and metastasis. Several exosomal contents have also been shown to promote metastasis and transfer metastatic ability to recipient cells [46, 81]. EMT is a crucial process that occurs before tumor metastasis, and it encompasses a complex process that includes cytoskeleton alterations, and downregulation of the expression of the adherens junction molecule E-cadherin. Exosomes derived from highly metastatic lung cancer cells and human late-stage lung cancer serum induce EMT and cause enhanced proliferation, migration, and invasion of non-cancerous recipient cells [38]. TGF-β enhances tumor invasion and metastasis by inducing EMT in lung cancer partly via interaction with circular RNAs (circRNAs). CircPTK2 and TIF1γ are significantly decreased in NSCLC cells undergoing TGF-β-induced EMT. However, circPTK2 overexpression augments the expression of TIF1γ, suppresses TGF-β-induced EMT, and inhibits NSCLC cell invasion [82]. Exosomes released by CAFs also promote EMT in lung cancer cells in a SNAI1-dependent manner [83]. Figure 3 provides an overview of factors associated with exosomal regulation to enhance lung tumor metastasis.

### Other sources of exosomes and lung cancer metastasis

Available data on the effect of MSCs on lung cancer cells is controversial with underlying mechanisms remaining unclear; this impedes the utilization of MSCs in tumor therapy. For instance, it has been reported that, although human umbilical cord MSC-conditioned medium (hucMSC-CM) promotes EMT, invasion, and migration, it also inhibits lung cancer cell proliferation and promotes their apoptosis. The EMT-promoting effect was mediated by hucMSC-derived exosomes, which were eliminated through inhibition of exosome release. Moreover, silencing TGF-β1 expression in the huMSCs reverted the EMT-promoting effect and enhanced the pro-apoptotic and anti-proliferative effects of hucMSCs on lung cancer cells via the exosomes, by deactivating Smad2/3, Akt/GSK-3β/β-catenin, NF-κB, extracellular-signal-regulated kinase (ERK), c-Jun N-terminal kinase (JNK), and p38 mitogen-activated protein kinase (MAPK) activated by TGF-β1 signaling [84]. The association between adipose-derived exosomes and tumor metastasis has been investigated. In one such study, the researchers demonstrated that adipocytes increase the invasive ability of lung tumor cells by secreting exosomes with a high concentration of MMP3, which in turn stimulates MMP9 activity in Lewis lung carcinoma (3LL) cells and promotes invasion in vitro and in vivo. Additionally, MMP3 protein levels in lung tumor tissues from obese patients are elevated, and positively correlate with MMP9 activity in lung tumor tissues [85].

Bone marrow-derived MSCs (BMMSCs) promote tumor growth and metastasis through paracrine-soluble cytokines or exosomes. BMSCs-derived exosomes cultured in hypoxic conditions are taken up by neighboring lung cancer cells and enhance cancer cell invasion and EMT. Exosome-mediated transfer of selected miRNAs, including miR-210-3p, miR-193a-3p, and miR-5100 from BMSCs to epithelial cancer cells activates STAT3 signaling and upregulates the expression of mesenchymal related molecules [86]. Platelets increase the tumor-promoting ability of BMMSCs by upregulating the protein levels of α-smooth muscle actin, vimentin, and fibroblast activation protein leading to trans-differentiation of BMMSCs into CAFs via TGF-β signaling. The resultant effect is BMMSCs-promoted tumor metastasis through enhanced proliferation and migration of tumor cells [87]. Table 1 presents some of the studies on the contribution of exosomes in lung cancer metastasis.

**Table 1:** Participation of exosomes in lung cancer metastasis

Source of exosome	Exosomal component	Mechanism or pathway	Principal effects	References
BMSCs	miR-210-3p, miR-193a-3p, and miR-5100	Exosome-mediated transfer of miRNAs activates STAT3 pathway	Enhanced cancer cell invasion and EMT	[86]
Melanoma	RAB1A, RAB5B, RAB7, and RAB27A	Reprogrammed cells were positive for c-Kit, receptor tyrosine kinase Tie2, and Met	Increased metastatic behavior, induced vascular leakiness at pre-metastatic sites, and reprogrammed bone marrow progenitor cells	[53]
Tumor	snRNAs	Activation of lung epithelial TLR3 to recruit neutrophils	TLR3-deficient mice showed reduced lung metastasis	[49]
		Phosphorylation of NF-κB p65 subunit and MAPKs ERK, JNK, and p38 signaling pathway	Exosomal snRNAs activated TLR3 to induce chemokine secretion and neutrophil recruitment	
hucMSCs	–	Activation of Smad2/3, AKT/GSK-3β/β-catenin, NF-κB, ERK, JNK, and p38 MAPK in TGF-β1 signaling pathways	Promoted EMT, invasion, and migration, yet inhibited proliferation and promoted apoptosis of lung cancer cells	[84]
Lung tumor	circSATB2	Positive regulation of FSCN1 expression via miR-326 in lung cancer cells	Enhanced proliferation, migration, and invasion of NSCLC cells, as well as an induced abnormal proliferation of normal human bronchial epithelial cells	[66]
Lung tumor	miR-660-5p	miR-660-5p targeting of KLF9 to promote tumorigenesis	Enhanced proliferation, migration, viability, and metastasis	[67]
Lung tumor	circ-CPA4 and let-7 miRNA	CPA4-regulated effects via let-7 miRNA/PD-L1 axis	Promoted cell stemness, mobility, and drug resistance of NSCLC	[68]
Lung tumor	lnc-MMP2-2	TGF-β-mediated exosomal miRNA regulation	Increased cancer cell migration, invasion potential, and vascular permeability	[69]
Mouse and human lung tumor	integrins α6β4 and α6β1	Activation of Src phosphorylation and pro-inflammatory S100 gene expression	Associated with lung metastasis and predict organ-specific metastasis	[50]
SCLC patients and mouse model	miR-141	Activation of miR-141/KLF12 pathway	SCLC angiogenesis	[42]
Administration of lung cancer-derived exosomes to NIH/3T3 cells	miR-210	Modulation of JAK2/STAT3 signaling pathway and TET2 in recipient fibroblasts	Increased angiogenesis via elevated expression of proangiogenic factors MMP9, FGF2 and VEGFA	[43]
NSCLC cells	miR-619-5p	Targeted inhibition of RCAN1.4	Promoted growth and metastasis of NSCLCs	[73]
Adipocytes	MMP3 and MMP9	Transfer of MMP3 to activate MMP9	Promoted lung tumor metastasis	[85]
Plasma	Three clusters of miRNAs including miR-574-5p, 328-3p and miR-423-3p	Regulation of the Wnt/β-catenin pathway	Involved in preconditioning the metastatic niche and promoting bone metastasis of lung cancer	[78]

## Clinical applications

Exosomal cargoes such as miRNAs and proteins are regarded as potentially ideal non‐invasive predictive tools for early diagnosis, prognosis, and therapeutic targets in lung cancer since they contain important information on signaling pathways associated with tumor biological responses. Their participation in conferring drug resistance to tumor cells also provides an avenue for clinical exploration and application.

### Drug resistance

Drug resistance is a phenomenon that occurs due to the adaptation of intracellular pathways or stimulation of survival-supporting paracrine and autocrine pathways, along with several expressed factors by drug-sensitive tumor cells following exposure to different therapies [88]. As key contributors to cell–cell communication, exosomes have been implicated in the induction of anti-tumor drug resistance [89, 90]. For instance, they transfer functional P-glycoprotein as they bind to drug-sensitive recipient cells, a key process in the induction of signal pathways necessary for drug resistance in recipient cells [91, 92]. Drug resistance to cisplatin, a platinum-based DNA damage drug administered intravenously to treat many cancers, has been demonstrated in NSCLC. Hypoxia exacerbated the drug resistance in lung cancer cells owing to upregulated expression of pyruvate kinase isozymes M2 (PKM2), which was observed in exosomes expressed by hypoxic cisplatin-resistance cells. Mechanisms associated with this process include enhanced glycolysis in NSCLC cells to generate reductive metabolites which neutralize cisplatin-induced reactive oxygen species (ROS), inhibition of apoptosis by exosomal PKM2 via a PKM2-BCL2-dependent manner, and reprogramming of CAFs to produce an acidic microenvironment that promotes NSCLC cell proliferation and cisplatin resistance [93].

Exosomes derived from lung cancer cells confer cisplatin resistance to other cancer cells and are associated with decreased expression of miR-100-5p which negatively regulates mammalian target of rapamycin (mTOR) expression [94]. Moreover, miR-206 regulates cisplatin resistance and EMT in human lung adenocarcinoma cells partly by targeting MET [95]. Certain serum exosomal RNAs such as miR-146a-5p may predict the efficacy of cisplatin for NSCLC patients, hence may represent a possible biomarker for real-time monitoring of drug resistance. In the event of cisplatin-induced drug resistance, the expression of miR-146a-5p gradually decreases in either NSCLC cells or the secreted exosomes. Mechanistically, miR-146a-5p increases the chemosensitivity of NSCLC cells to cisplatin by targeting Atg12 to inhibit autophagy [96]. Other studies of lung cancer drug resistance include tumor release of lncRNA H19 which promotes resistance to gefitinib by packaging into exosomes [97] and exosome-mediated transfer of lncRNA RP11‑838N2.4 which enhances erlotinib resistance [98].

### Predictive value

Exosomes have been employed to effectively differentiate lung cancer patients from healthy individuals. Specifically, exosomal components such as RNAs and proteins continue to be explored as promising diagnostic and prognostic biomarkers.

#### Diagnostic biomarkers

In addition to the contribution of exosomal miR-210-3p, miR-193a-3p, and miR-5100 to lung cancer cell invasion and EMT, the diagnostic efficacy of these individual miRNAs indicates that plasma exosomal miR-193a-3p can significantly differentiate cancer patients from non-cancerous controls. However, a panel of these three plasma exosomal miRNAs exhibited a better diagnostic accuracy in discriminating lung cancer patients with or without metastasis than individual exosomal miRNAs [86]. A study has shown that miR-378 is differentially expressed in NSCLC of patients with brain metastasis, and may be a potential biomarker for characterizing NSCLC brain metastasis. Further miR-378 levels have also aided clinicians in stratifying high-risk patients on a clinical trial for either prophylactic cranial irradiation or a new intervention that may abrogate brain metastasis development, ultimately causing a new standard of care for NSCLC patients [72]. Similarly, serum miR-330-3p levels are higher in NSCLC patients with brain metastasis than those without metastasis. The underlying mechanism indicates that miR-330-3p targets GRIA3 to mediate the interaction between GRIA3-TGF-β1, resulting in EMT, tumor proliferation, migration, and invasion. This suggests miR-330-3p may be a potentially useful biomarker for identifying NSCLC patients with metastatic potential [80]. A study evaluating the differential expression of miRNAs in the serum of patients with NSCLC reported that miR-15a-5p, miR-25-3p, miR-320a, miR-192-5p, let-7d-5p, let-7e-5p, miR-92a-3p, miR-148a-3p, but also miR-10b-5p and miR-375 were significantly downregulated in the serum of NSCLC patients and lung squamous cell carcinoma patients, respectively. Although none of the miRNAs correlated with the therapeutic response or survival outcomes, the expression of miR-25-3p, miR-320a, and miR-148a-3p significantly correlated with the lung cancer stage [99]. Exosomal miR-106b levels were found to be much higher in the serum of lung cancer patients than in healthy individuals and was linked with TNM stages and lymph node metastasis. Moreover, miR-106b enhanced the expression of MMP-2 and MMP-9 and targeted PTEN to promote lung cancer cell migration and invasion [100]. This presents exosomal miR-106b as both a promising diagnostic biomarker and a possible drug target for patients with lung cancer.

Exosomal proteins have also been assessed as potential biomarkers for lung cancer. Serum exosomal analysis for lung cancer-related proteins in 109 NSCLC patients with advanced-stage (IIIa-IV) disease and 110 matched control subjects, generated a comprehensive 30-marker model which differentiated the two groups at an accuracy rate of 75.3%. CD317 and EGFR were highly expressed on the exosomal surface and could be reliable biomarkers for diagnosing NSCLC [101]. Leucine-rich α-2-glycoprotein (LRG1) is found to be highly expressed in urinary exosomes and lung tissue of NSCLC patients, indicating it potential as a candidate biomarker [102]. The exosomal markers CD151, CD171, and tetraspanin 8 were identified as the strongest differentiators of patients with lung cancer of all histological subtypes from patients without lung cancer. In squamous cell cancer and SCLC, multimarker models were not superior to CD151 as an individual marker in differentiating cancer patients from non-cancer patients [103]. These findings present exosome protein profiling as a promising diagnostic tool in lung cancer.

#### Prognostic biomarkers

The prognostic value of FLI1 exonic circular RNA (FECR), as a new malignant driver that determines the metastatic phenotype in SCLC, has been assessed. It was shown that CRISPR Cas9 knockout and shRNA knockdown of FLI1 identified FECRs as a new noncanonical malignant driver in SCLC, as FECR1 (exons 4-2-3) and FECR2 (exons 5-2-3-4) were aberrantly upregulated in SCLC tissues, and positively correlated with lymph node metastasis. Notably, the serum concentration of exosomal FECR1 was associated with poor survival and clinical response to chemotherapy, making FECR1 a promising prognostic biomarker to track disease progression of lung cancer [104]. Other studies have reported significantly upregulated levels of FLI1 in small cell lung cancer (SCLC) tissues compared to NSCLC and normal lung tissues. The expression of FLI1 oncoprotein positively correlates with the extensive stage of SCLC and overexpressed Ki67, a nuclear marker that is closely related to tumor cell proliferation. Mechanistically, FLI1 promotes tumorigenesis by activating the miR-17-92 cluster family [105] and miR584-ROCK1 [104] pathways. Considering the reported involvement of long intergenic non-protein coding RNA 680 (LINC00680) in various cancers, Wang et al. investigated its effects in lung cancer. The study reported that LINC00680 is upregulated in NSCLC and is closely associated with malignancy and the poor prognosis of NSCLC patients. LINC00680 enhanced the growth of NSCLC cells in mice, increased proliferation and colony formation, and suppressed apoptosis of H1299 and A549 cells, by functioning as a sponge of miR-410-3p to improve HMGB1 expression [106].

Several studies have proven that tumor growth and metastasis greatly contribute to poor prognosis in patients with NSCLC through pathological angiogenesis that produces new abnormal and poorly organized vessels based on a pre‐existing vascular network [107, 108]. A study examined the predictive value of angiogenic miRNAs for disease‐free survival (DFS) and overall survival (OS) of patients with NSCLC. The results showed a median DFS of 30.0 (14.0–49.0) months, and a median OS of 41.5 (23.0–58.0) months, with the 5‐year DFS and OS rates being 11.3% and 32.3%, respectively. Further analysis indicated that high plasma concentrations of miR‐18a, miR‐20a, miR‐92a, miR‐210, and miR‐126 correlated with poor prognosis of lung cancer patients. Moreover, increased expression of plasma miR‐18a, miR‐20a, and miR‐92a, as well as in lymphatic nodes, was identified as an independent risk factor for both DFS and OS in NSCLC patients [109]. Evaluation of the prognostic value of circulating angiopoietin-2 (Ang-2) mRNA levels before treatment of NSCLC patients has been documented. Patients with a high concentration of circulating Ang-2 mRNA have diminished OS which even worsens in patients with stage IV lung cancer [110]. Tumor-derived exosomal RNA eIF4E in serum was proposed as a practical tool to predict the prognosis of NSCLC. Lung cancer patients with higher exosomal eIF4E expression were more likely to present distant metastasis, advanced TNM stage, and serum positive cytokeratin fragment 19 (CYFRA21-1), and exosomal eIF4E was also determined to be an independent prognostic factor for shorter OS and progression-free survival [111]. The application of exosomal components in the diagnosis and prognosis of lung cancer are summarized in Table 2.

**Table 2:** Predictive utility of exosomes in lung cancer

Experimental model/source	Exosomal component	Predictive effect	References
Mouse syngeneic tumor model	miR-210-3p, miR-193a-3p, and miR-5100	miR-193a-3p alone can differentiate cancer patients from non-cancerous controls, but the combination of the three gives better diagnostic accuracy	[86]
Primary SCLC tissues and NSCLC tissues	FLI1 exonic circular RNAs	Serum exosomal FECR1 is associated with poor survival and clinical response to chemotherapy	[104]
Human NSCLC tissue	miR‐18a, miR‐20a, miR‐92a, miR‐210, and miR‐126	Prognostic biomarkers in patients with NSCLC—poor prognosis	[109]
NSCLC patients	eIF4E	Independent prognostic factor for shorter overall survival and progression-free survival	[111]
Lung cancer patients’ serum	miR-574-5p, 328-3p and miR-423-3p	Deferential in lung cancer patients with bone metastasis	[78]
NSCLC patients	miR-330-3p	Biomarker for identifying NSCLC with metastatic potential, especially brain metastasis	[80]
Serum of NSCLC patients and lung squamous cell carcinoma of patients	miR-15a-5p, miR-320a, miR-25-3p, miR-192-5p, let-7d-5p, let-7e-5p, miR-148a-3p, miR-92a-3p, miR-375 and miR-10b-5p	Deferential in lung cancer patients and correlates with cancer stage	[99]
NSCLC cells	miR-34c-3p	Diagnostic and prognostic marker for NSCLC with low levels indicating tumor invasion and migration	[116]
Serum of lung cancer patients	miR-106b	Promising diagnostic biomarker and drug target for patients with lung cancer	[100]
Serum of lung cancer patients	CD317 and EGFR	Diagnostic biomarker of NSCLC	[101]
Urine and lung tissue of NSCLC patients	LRG1	Diagnostic biomarker of NSCLC	[102]
Plasma of lung cancer patients	CD151, CD171, and tetraspanin 8	Diagnostic biomarker of lung cancer	[103]

### Therapeutics

Available conventional therapies for lung cancer are not effective for metastatic lung cancer treatment. Considering the critical role played by exosomes in the tumor microenvironment, studies continue to explore exosomal targets as a possible novel therapeutic strategy for cancer. In this quest, exosomal components that suppress tumor growth are targeted to increase their upregulation while tumor-promoting cargoes are inhibited. Moreover, exosomes could also be engineered or modified to deliver certain active molecules to target sites or express desired molecules with therapeutic effects.

#### Tumor-suppressive exosomal components

Several studies have reported the tumor-suppressive effect of exosomal components, including oncogenic miRNAs and tumor suppressor miRNAs. For instance, miR-302b is known to inhibit cancer progression by targeting oncogenes post-transcriptionally. Mechanistically, the exosomes-derived miR-302b suppresses lung cancer cell proliferation and migration through the TGF-βRII/ERK pathway, thus may represent a potential target for human lung cancer therapy [112]. Pigment epithelium-derived factor (PEDF) is an anticancer protein that modulates lung cancer progression and metastasis; thus, regulating cancer cell-derived exosomal secreted factors. According to Huang et al., PEDF inhibits the metastatic potential of lung cancer cells by increasing thrombospondin 1 release in cancer cell-derived exosomes, leading to suppressed cytoskeletal remodeling and exosome-induced lung cancer cell motility, migration, and invasion [113]. Exosomal pretreatment with PEDF produces antitumor effects including enhanced autophagy and activation of cancer cell apoptotic pathways [114, 115], hence a promising therapy. CircPTK2 and TIF1γ are significantly downregulated in NSCLC cells undergoing TGF-β-induced EMT. Further analysis revealed that circPTK2 (hsa_circ_0008305) could suppress TGF-β-induced EMT and metastasis through the regulation of TIF1γ in NSCLC [82], presenting a novel mechanism by which circRNA modulates TGF-β-induced EMT and lung cancer metastasis, and suggesting that targeted overexpression of circPTK2 could provide a therapeutic strategy for advanced NSCLC.

#### Tumor-promoting exosomal components

Exosomal circular RNA circ-CPA4 is known to interact with NSCLC cells derived intracellular and extracellular programmed cell death ligand PD-L1 to enhance lung cancer progression and drug resistance, and facilitate tumor immune evasion. It has been demonstrated that knock-down of circ-CPA4 effectively prevents this occurrence as evidenced by inhibited cell growth, cell mobility, and EMT, and increased cell death in lung cancer as PD-L1 is downregulated by serving as an RNA sponge for let-7 miRNA [68]. FECRs promote lung cancer malignancy metastasis and reduced response to chemotherapy through FECRs sequestration and subsequent inactivation of tumor suppressor miR-584-3p, leading to the activation of the Rho Associated Coiled-Coil Containing Protein Kinase 1 gene (ROCK1). The inhibition of FECRs significantly reduces the migration in two highly aggressive SCLC cell lines and decreased tumor metastasis in vivo [104]. Lung cancer-derived exosomes carrying low levels of miR-34c-3p could be shuttled into the cytoplasm of NSCLC cells, and accelerate NSCLC migration and invasion by upregulating integrin α2β1. Furthermore, integrin α2β1 is a direct target of miR-34c-3p, and the upregulation of integrin α2β1 could encourage lung tumor cell invasion and migration. Serum-derived exosomes from NSCLC patients showed significantly reduced expression of miR-34c-3p compared to normal individuals [116]. Tripartite motif-containing 59 (TRIM59) is expressed in lung cancer cell-derived exosomes and could be transferred to macrophages via exosomes, leading to macrophage activation, which in turn promotes lung cancer progression. The mechanism underlying this process indicates that tumor-derived exosomal TRIM59 triggers macrophages to exhibit tumor-promoting activities by modulating abhydrolase domain containing 5 (ABHD5) proteasomal degradation, which activates the NLRP3 inflammasome signaling pathway and promotes lung cancer progression by IL-1β secretion [117]. Inhibiting VEGFR1 function through the use of antibodies or by the removal of VEGFR1(+) cells from the bone marrow of wild-type mice abrogates the formation of pre-metastatic clusters and prevents tumor metastasis [52].

#### Engineered exosomes

In recent years, engineered exosomes have attracted growing research attention as drug delivery carriers for cancer treatment. This is partly because of their distinctive property of organotropism, which plays a crucial role in organ distribution following systemic administration [118, 119]. Nie et al. designed lung-specific exosomes (231-Exo) for miRNA-126 delivery into lung cancer. They observed that the miRNA-126 loaded exosome effectively escaped immune surveillance, and significantly inhibited A549 lung cancer cell proliferation and migration via the interruption of the PTEN/PI3K/AKT (phosphatase and tensin homolog/phosphatidylinositol 3-kinase/protein kinase B) signaling pathway. Further analysis in a lung metastasis mouse model showed that the miRNA-231-Exo effectively targeted the lung and produced an effective suppression of lung metastasis formation [120]. Based on a previous study that showed that soluble FMS-like tyrosine kinase-1 (sFlt-1) exerts anti-tumor activity by suppressing angiogenesis in many cancers, exosomes have been used to load sFlt-1 and tested in both in vitro and in vivo as potential lung cancer therapy. The therapeutic formulation resulted in higher inhibition efficacy on pro-angiogenesis, significant anti-tumor activity via the inhibition of the growth of NCI-H69 tumor xenografts, increased tumor apoptosis, and inhibition of tumor cell proliferation in mice [121]. Researchers have engineered a drug delivery system consisting of nanosomes, by conjugating gold nanoparticles (GNPs) with the anticancer drug doxorubicin (Dox) and then linking these to the exosome pH-sensitive hydrazone. The resultant complex showed stable cell viability with the constructed nanosomes exhibiting preferential cytotoxicity against cancer cells [122].

Anthocyanidins have also been encapsulated into exosomes and applied in the treatment of multiple tumors, including lung cancer in nude mice with enhanced therapeutic effects [123]. Another study engineered and optimized a formulation comprising exosomes loaded with the potent anti-cancer agent paclitaxel that incorporated the aminoethylanisamide-polyethylene glycol (AA-PEG) vector moiety to target the sigma receptor overexpressed by lung cancer cells. The engineered exosomal complex exhibited a high loading capacity, profound ability to accumulate in cancer cells upon systemic administration, and improved therapeutic outcomes [124]. Pretreatment of exosomes with Sanguinarine, a benzo[c]phenanthridine alkaloid obtained from the roots of *Sanguinaria canadensis,* effectively averted the effects of lung cancer-derived exosomes, including tumor cell proliferation, invasion, migration activities, and suppressed apoptosis via inhibition of macrophages and the NF-κB pathway [125].

## Discussion and conclusion

Lung cancer metastasis is a crucial phase of tumor progression and targets mainly the brain, bone, adrenal gland, and liver, and is a major cause of lung cancer mortality. NSCLC accounts for over 80% of lung cancer cases and has an overall five-year survival rate of only 15%. Patients who present with advanced-stage NSCLC die within 18-months of diagnosis. Over 70% of these mortalities are attributable to metastatic spread of the lung tumor, thus elucidation of the underlying mechanistic basis of lung cancer metastasis would have a significant impact on patient quality of life and survival. Recent studies have provided evidence to the effect that, the formation of tumor-promoting pre-metastatic niches in secondary organs adds a previously unnoticed level of complexity to the undertaking of treating patients with metastatic diseases including lung cancer. In this context, primary tumor cells orchestrate pre-metastatic niche formation by secreting a variety of growth factors and cytokines that favor the mobilization and recruitment of bone marrow-derived cells to future metastatic sites [126]. The secretion of tumor-derived exosomes and induction of hypoxia within the primary tumor continue to emerge as crucial vehicles and processes, respectively, for tumor-derived factors to regulate pre-metastatic sites. It has also been demonstrated that reduced immune surveillance is a novel mechanism by which primary tumors produce favorable niches in secondary organs.

The inadequacy of current diagnostic techniques for lung cancer hampers the ability of early detection of lung cancer, resulting in increased mortality and failure of available therapeutic approaches. This calls for the conduct of in-depth studies that stratify the various stages of lung cancer progression, for the focused discovery of biomarkers that can effectively predict early diagnosis, prognosis, and treatment efficacy. Interestingly, tumor-derived exosomes have been shown to be involved in the formation of not only the tumor but also the pre-metastatic niche at the site of future metastasis and encourages the growth of disseminated lung tumor cells. Exosomal miRNAs and proteins play an important role in the regulation of a variety of targets and, consequently multiple pathways, which make them a powerful tool for early detection of disease, risk assessment, and prognosis. The application of non-invasive techniques such as blood-based methods in the identification of biomarkers is of utmost significance for early diagnosis and predictive prognosis of advanced-stage lung cancer patients. Continuous pursuit of the promising effects of exosomes in lung cancer for future clinical application is a hopeful quest in the right direction.

Moreover, engineering exosomes as carriers of therapeutic agents is emerging as promising drug delivery system, specifically in anti-tumor high precision medicine due to their special biological properties such as intrinsic intercellular communication ability, outstanding biocompatibility, low toxicity and immunogenicity, long blood circulation capability, biodegradable characteristics, and ability to cross various biological barriers and homing to the target site [3, 9]. Regardless, it is highly essential to further explore other characteristic properties of exosomes, such as tumor specificity, biomolecular transport and loading capacity, circulatory stability, among others to validate their contribution to lung cancer treatment.

In future, insight into the mechanisms of distinct exosomal proteins and RNA packaging, as well as specific exosome biogenetic pathways active in cancer cells, is highly necessary. Because exosomal cargoes are unique, research into their high selectivity for cancer cells and associated microenvironment could uncover tumor-specific pathways and molecules for more efficient cancer prediction and therapeutic targeting. Notwithstanding, the current knowledge of the role of exosomes as presented here highlights an array of exosome-dependent pathways and cargoes that are ripe for exploitation as therapeutic targets to treat lung cancer metastasis, and for predictive value assessment in diagnosis, prognosis, and anti-tumor drug resistance.

## Data Availability

Not applicable.

## Abbreviations

BMSCs: Bone marrow mesenchymal stem cell
EMT: Epithelial mesenchymal transition
RNAs: Ribonucleic acids
mRNA: Messenger RNA
miRNA: MicroRNA
DNA: Deoxyribonucleic acid
STAT3: Signal transducer and activator of transcription 3
snRNAs: Small nuclear RNAs
hucMSCs: Human umbilical cord mesenchymal stem cell
lnc-MMP2-2: Long noncoding- matrix metalloproteinase 2
TET2: Ten-eleven translocation 2
NSCLC: Non-small cell lung cancer
SCLC: Small cell lung cancer
FLI1: Friend leukemia virus integration 1
Treg: Regulatory T-cells
MDSCs: Myeloid-derived suppressor cells
NK: Natural killer
PD-L1: Programmed death-ligand 1
IFN-γ: Interferon-γ
TGF-β1: Tissue growth factor-β1
EGFR: Endothelial growth factor receptor
DCs: Dendritic cells
VEGF: Vascular endothelial growth factor
FGF: Fibroblast growth factors
IL: Interleukin
HUVECs: Human umbilical vein endothelial cells
GAS5: Growth arrest specific 5
PTEN: Phosphatase and tensin homolog
PI3K: Phosphatidylinositol 3-kinases
CAFs: Cancer-associated fibroblasts
MMP-9: Matrix metalloproteinase 9
JAK2: Janus kinase 2
STAT: Signal transducer and activator of transcription
MORC2: MORC family CW-type zinc finger 2
TLR: Toll-like receptor
CXCL: Chemokine [C-X-C motif] ligand
TNFα: Tumor necrosis factor α
NFκB: Nuclear factor-κ-B
EGFR: Epidermal growth factor receptor
RANKL: Receptor activator of nuclear factor kappa-Β ligand
CXCR4: C-X-C chemokine receptor type 4
VCAM1: Vascular cell adhesion molecule 1
ERK: Extracellular-signal-regulated kinase
JNK: C-Jun N-terminal kinase
MAPK: Mitogen-activated protein kinase
mTOR: Mammalian target of rapamycin
